# Effect of I-Phase on Microstructure and Corrosion Resistance of Mg-8.5Li-6.5Zn-1.2Y Alloy

**DOI:** 10.3390/ma16083007

**Published:** 2023-04-10

**Authors:** Ziming Fang, Liangxu He, Jiaxiu Wang, Xiaochun Ma, Guixiang Wang, Ruizhi Wu, Siyuan Jin, Jiahao Wang, Zihui Lu, Zhenzhao Yang, Boris Krit, Sergey Betsofen, Iya I. Tashlykova-Bushkevich

**Affiliations:** 1 Key Laboratory of Superlight Materials & Surface Technology, Ministry of Education, Harbin Engineering University, Harbin 150001, China; 2 Moscow Aviation Institute, National Research University, 125993 Moscow, Russia; 3 Physics Department, Belarusian State University of Informatics and Radioelectronics, 220013 Minsk, Belarus

**Keywords:** Mg-Li alloys, solid solution treatment, microstructure, corrosion performance, I-phase

## Abstract

The effects of solid solution treatment duration on the corrosion behavior and microstructure behavior of the cast Mg-8.5Li-6.5Zn-1.2Y (wt.%) alloy were investigated. This study revealed that with the treatment time for solid solutions increasing from 2 h to 6 h, the amount of α-Mg phase gradually decreases, and the alloy presents a needle-like shape after solid solution treatment for 6 h. Meanwhile, when the solid solution treatment time increases, the I-phase content drops. Exceptionally, under 4 h of solid solution treatment, the I-phase content has increased, and it is dispersed uniformly over the matrix. What we found in our hydrogen evolution experiments is that the hydrogen evolution rate of the as-cast Mg-8.5Li-6.5Zn-1.2Y alloy following solid solution processing for 4 h is 14.31 mL·cm^−2^·h^−1^, which is the highest rate. In the electrochemical measurement, the corrosion current density (*i*_corr_) value of as-cast Mg-8.5Li-6.5Zn-1.2Y alloy following solid solution processing for 4 h is 1.98 × 10^−5^, which is the lowest density. These results indicate that solid solution treatment can significantly improve the corrosion resistance of the Mg-8.5Li-6.5Zn-1.2Y alloy. The I-phase and the α-Mg phase are the primary elements influencing the corrosion resistance of the Mg-8.5Li-6.5Zn-1.2Y alloy. The existence of the I-phase and the border dividing the α-Mg phase and β-Li phase easily form galvanic corrosion. Although the I-phase and the boundary between the α-Mg phase and β-Li phase will be corrosion breeding sites, they are more effective in inhibiting corrosion.

## 1. Introduction

The Mg-Li alloy, due to its light weight, high specific strength, effective electromagnetic shielding, and excellent damping performance, is considered to be one of the most advanced materials with broad opportunities for application in fields such as aerospace, weapons, and electrical apparatuses [1,2,3,4,5]. As far as the Mg-Li alloy is concerned, the crystal structure changes with the addition of a Li element [6,7,8]. When the content of Li does not exceed 5.7 wt.%, the alloy is completely composed of the α-Mg phase with the hexagonal close-packed (HCP) structure; when Li content is in the range of 5.7 to 10.3 wt.%, the alloy is composed of the α-Mg phase and the body-centered cubic (BCC) structured β-Li phase; when the Li content exceeds 10.3 wt.%, the alloy consists of the β-Li phase [9,10,11]. As a hot research topic, the Mg-8Li alloy with a dual-phase structure has excellent comprehensive properties. However, as a result of the intense chemical activity, with the increase of element Li in the alloy, the corrosion resistance will decline gradually [12]. Meanwhile, in the duplex phase Mg-Li alloy, due to the potential difference between the α-Mg phase and the β-Li phase in the Mg-Li alloy, galvanic corrosion occurs readily, which means that the duplex phase Mg-Li alloy possesses poorer corrosion resistance than single phase Mg-Li alloys [13]. Therefore, it is essential to research corrosion and protection of the dual-phase Mg-Li alloy.

At present, alloying [14,15], heat treatment [16,17], severe plastic distorting [9,18,19], and surface coating [20,21,22] are commonly used to improve the corrosion resistance of the Mg-Li alloy. Alloying can effectively enhance the corrosion performance of the Mg-Li alloy. Generally, Al, Zn, Zr, Mn, rare earth elements, etc. are added into Mg-Li alloys to increase the corrosion resistance by forming a second phase in the matrix or by changing the composition of the surface oxide film [23]. Rare earth elements can also combine with Fe, Ni, Co, and other impurities to form intermetallic compounds which improve corrosion resistance [15]. The icosahedral phase (I-phase) has a unique rotational symmetry structure that is different from the traditional crystal, so it has excellent performance. It has also been reported that the icosahedral quasicrystal phase (I-phase) can be formed in the Mg-Zn-Y alloy by controlling the atomic ratio of Zn/Y between 4.4 and 7, thus significantly improving the mechanical properties and corrosion resistance of the alloy [24,25]. Xu et al. found that the addition of Zn and Y could form the I-phase in the Mg-6Li alloy, which greatly improved the alloy’s corrosion resistance [26]. In the Mg-Li alloy, the I-phase can form a stable eutectic structure with the α-Mg phase; meanwhile, the I-phase distributes at the border, dividing the α-Mg phase and the β-Li phase [27]. The introduction of the I-phase into Mg-Li alloys not only improves corrosion resistance, but also refines the grain size and improves the mechanical properties of the alloy [28], hence the Mg-Li alloy with I-phase has been widely considered [24].

In the Mg-Li alloy, due to the negative electrode potential of the α-Mg phase and the β-Li phase, the electrode potential of impurity phases formed during alloy melting are generally higher than that of matrix phases, thus forming galvanic corrosion with matrix phases [29]. Among them, the impurity phases with high potential are protected as a cathode, and the matrix phases with low potential are corroded as an anode [30]. At the same time, the defects and segregation in the as-cast structure also have a detrimental outcome on the corrosion performance. The heat treatment can not only get rid of the residual stress and some defects of the castings, but also tailor the microstructure and element distribution. According to the literature, heat treatment can also effectively improve the strength and low cycle life of magnesium alloys [31,32]. Solution treatment of the Mg–9Li alloy at 1067 K for 4.5 h increased its yield strength from 62 to 110 MPa; the UTS increased from 100 to 120 MPa, and elongation increased from 32% to 45% [33]. Therefore, heat treatment is an effective way to improve the corrosion resistance and mechanical properties of the alloy. Wang et al. [34] found that the MgZn_2_ phase in the Mg-6.7%Zn-1.3%Y-0.6%Zr alloy can be dissolved, and nanoscale I-phase precipitates formed in solid solution treatment improve the corrosion resistance and improve the strength of the alloy. Wan et al. [35] researched how corrosion properties are affected by the solid solution treatment of the Mg_97_Y_2_Zn_1_ alloy, and learned that after solid solution treatment, the I-phase in the alloy was decomposed. At the same time, the continuous network structure is transformed into a granular structure, which increases the resistance to corrosion. When Liu et al. [36] conducted solid solution treatment at 500 °C for the Mg_82_Zn_14.2_Y_3.3_Zr_0.5_ alloy, a longer time was provided for the formation of the I-phase because the alloy was in a semi-melted state, and the movement of each atom was less restricted, which greatly increased the I-phase in the alloy. Considering the aforementioned details, the corrosion resistance of the Mg-Li alloy containing the I-phase can possibly be improved by solid solution treatment. However, it has not received much attention.

In this work, it is suggested that the corrosion resistance of the Mg-8.5Li-6.5Zn-1.2Y alloy containing I-phase should be studied. The effects of different solid solution treatment times on the surface morphology and spatial distribution of the α-Mg phase and I-phase, as well as their effects on corrosion performance, were investigated. 

## 2. Experimental Procedures

### 2.1. Material Preparation

The Mg-8.5Li-6.5Zn-1.2Y alloy used in the experiment was smelted in a vacuum electromagnetic induction furnace under the protection of pure argon, and its raw materials were commercial pure Mg ingots (99.9 wt.%), commercial pure Li ingots (99.9 wt.%), commercial pure Zn ingots (99.9 wt.%), and Mg-20Y (wt.%) master alloy. The ingots were divided into plates of 40 mm × 40 mm × 10 mm. 

### 2.2. Heat Treatments

The plates were solid solution treated at 500 °C for 2 h, 4 h, and 6 h, respectively. Then, the removed samples were quenched in cold water at room temperature; the volume of cooling water used was 5 L. In order to prevent the plates from burning during the heat treatment, the plates were buried in a crucible filled with MgO powder after being coated in Al foil.

### 2.3. Characterization

#### 2.3.1. Microstructures and Phase Compositions

The samples used to observe microstructure were ground with SiC papers progressively down to 2000 grit and mechanically polished. Then, they were observed by an optical microscope (OM, LEICA DMIRM), and scanning electron microscope (SEM, Thermoscientific Apreo S Lovac) coupled with an energy dispersive spectrometer (EDS, the model used is compatible with scanning electron microscopy). The phase composition of the samples was established by X-ray diffractometer (XRD, Rigaku TTR-III) after grinding with 800 grit SiC paper. The scanning range was 20–80° with a speed of 5°/min.

#### 2.3.2. Corrosion Assessed

The samples used in the hydrogen evolution (a hydrogen evolution reaction is the production of hydrogen through the process of water electrolysis) experiment were divided into a size of 20 mm × 20 mm × 10 mm, and only one side was exposed by wrapping with epoxy resin. Then, samples were ground on SiC papers progressively down to 2000 grit and mechanically polished. The hydrogen analysis experiment uses an inverted funnel with a burette attached to it to collect hydrogen gas produced during corrosion for measurement. At room temperature, the samples were submerged in 3.5 wt.% NaCl solution and the hydrogen generated within 72 h was collected to calculate the hydrogen evolution rate. NaCl was chosen as the corrosion medium in the simulated marine environment. The size of the samples used in the immersion tests was 10 mm × 10 mm × 10 mm, and one side of the sample was ground up to 3000 grit with SiC papers and mechanically polished. The pre-treated samples were soaked in 3.5 wt.% NaCl solution for 2 h at room temperature, then the corrosion products were removed by immersion in a 180 g/L CrO_3_ chromic acid solution for 1 min [9]. Finally, digital camera and scanning electron microscope (SEM) were used to observe the macro and micro corrosion morphology and clarify the corrosion mechanism. The size and pretreatment of samples for the mass loss test are consistent with those for the hydrogen evolution test; then, they were immersed in 3.5 wt.% NaCl solution at room temperature for 48 h. With an accuracy of 0.1 mg, the weight of the samples was measured both before and after immersion using an electronic balance scale. For each condition, three parallel samples were used to determine the changes in the measured data.

Using an electrochemical workstation (Zahner, IM6), the alloy’s corrosion resistance was assessed in a 3.5 wt.% NaCl solution. A classical three-electrode cell with an exposed area of 20 mm × 20 mm and a counter electrode of Pt foil, reference electrode of saturated calomel electrode (SCE), and working electrode of sample surfaces was employed. Before the test, the test area of the samples was ground up to 3000 grit with SiC papers and mechanically polished. The potentiodynamic polarization curves were measured in the open circuit potential ±300 mV range at a scan rate of 1 mV·s^−1^. Electrochemical impedance spectroscopy (EIS) was measured on the samples in the frequency range of 100 kHz–10 mHz, with an amplitude of 5 mV [37]. Experimental data were fitted with CorrView software and ZView. To make sure of the reliability of the experiment, each experiment group underwent three tests.

The size of the samples used in the immersion tests was 10 mm × 10 mm × 10 mm, and one side of the sample was ground up to 3000 grit with SiC papers and mechanically polished. The pre-treated samples were soaked in 3.5 wt.% NaCl solution for 2 h at room temperature, then the corrosion products were removed by immersion in a 180 g/L CrO_3_ chromic acid solution. Finally, scanning electron microscope (SEM) was used to observe the corrosion morphology and clarify the corrosion mechanism. 

## 3. Results and Discussion

### 3.1. Phase Compositions and Microstructures of Mg-8.5Li-6.5Zn-1.2Y Alloy

**Figure 1:**
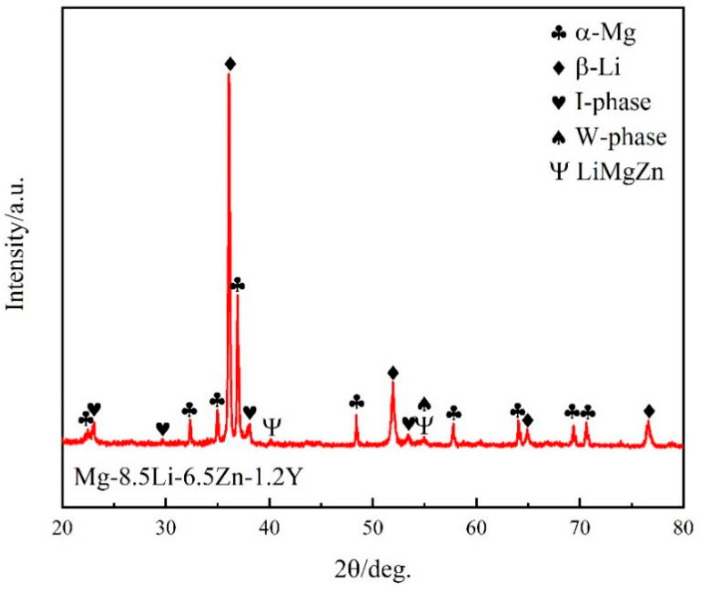
XRD pattern of the as-cast Mg-8.5Li-6.5Zn-1.2Y alloy.

The Mg-8.5Li alloy is composed of the α-Mg and β-Li phases [38]. However, for the as-cast Mg-8.5Li-6.5Zn-1.2Y alloy, Figure 1 shows its XRD pattern. The results indicate that in addition to the α-Mg and β-Li phases, there are also I-phase, W-phase (Mg_3_Zn_3_Y_2_), and LiMgZn phase in the alloy. Simultaneously, it is evident from the figure that the peaks of the W-phase and LiMgZn phase are low, indicating that most of the phase generated due to the addition of Zn and Y elements is the I-phase.

**Figure 2:**
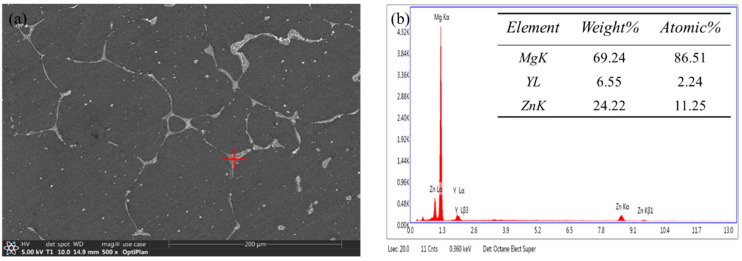
The SEM image of the as-cast Mg-8.5Li-6.5Zn-1.2Y alloy (**a**) and EDS analysis of I-phase (**b**).

Figure 2 shows the SEM image of the as-cast Mg-8.5Li-6.5Zn-1.2Y alloy. An abundance of the network’s second phases is distributed in the alloy matrix, as shown in Figure 2a. By EDS analysis, the atomic ratio of Zn/Y is 5.02, which proves that the network phases are I-phase, as shown in Figure 2b. The EDS of the I-phase did at least ten point tests to get the average value. During EDS measurement, the voltage selected was 20 kV, under which the depth measured by the primary electron beam was 3–5 microns. In the figure, the second phase was distributed along the crystal and its size was about 10 microns, so it can be determined that the element composition of the second phase was measured by EDS.

**Figure 3:**
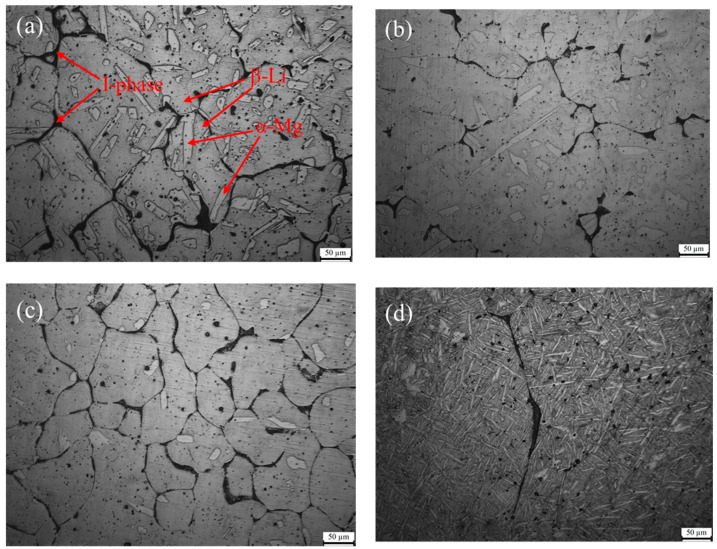
Optical microstructure of the Mg-8.5Li-6.5Zn-1.2Y alloys with different solid solution treatment times (**a**) as-cast, (**b**) 2 h, (**c**) 4 h, and (**d**) 6 h, respectively.

**Figure 4:**
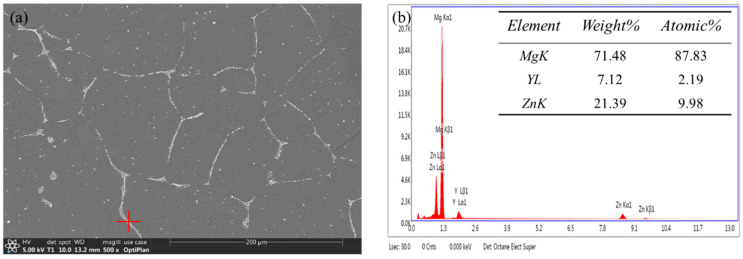
The SEM image of the solid solution treated Mg-8.5Li-6.5Zn-1.2Y alloy for 4 h (**a**) and EDS analysis of I-phase (**b**).

The morphology and distribution of the α-Mg phase and I-phase in the Mg-8.5Li-6.5Zn-1.2Y alloy changed after being solution treated for different times, as shown in Figure 3a–d. The as-cast Mg-8.5Li-6.5Zn-1.2Y alloy is mainly composed of the α-Mg phase, β-Li phase, and I-phase, as shown in Figure 3a. The I-phase is distributed in an inhomogeneous network structure in the matrix. In addition, it can also be seen that there are many granular impurities in the matrix. Through subsequent experimental analysis, we concluded that the impurity particles are the Mg_3_Zn_2_ phase. After the sample was solid solution treated for 2 h, the continuous I-phase in the sample is fused and the volume fraction decreased significantly; furthermore, the agglomerated I-phase changed to be uniform. Meanwhile, the impurities in the matrix are reduced, which proves that the I-phase and some impurities have been partially dissolved into the matrix, as shown in Figure 3b. After the sample was solid solution treated for 4 h, as shown in Figure 3c, many elongated second phases precipitate along the grain boundary, making the grain boundary clearly visible. EDS analysis for this second phase shows that the atomic ratio of Zn/Y is 4.56 and it can be determined to be the I-phase, as shown in Figure 4a,b. Therefore, compared with the solid solution treatment for 2 h, the amount of I-phase in the sample with a solid solution treatment for 4 h increases. This occurs because, with the extension of solid solution treatment time, the α-Mg phase decreases and spheroidization occurs after the solution time reaches 4 h, and the I-phase transition becomes more uniform. At the same time, many elongated second phases precipitate along the grain boundary, making the grain boundary clearly visible. EDS analysis confirms that the elongated second phase is I-phase, as shown in Figure 2. When the solid solution time is 6 h, almost all the I-phase is dissolved into the matrix, as shown in Figure 3d. According to previous studies, the Mg-8.5Li-6.5Zn-1.2Y alloy has become a supersaturated solid solution. It is evident that the α-Mg phase changes in morphology, which is densely distributed in the alloy matrix with a needle shape. Guo et al. [39] reported that severe deformation at room temperature can lead to the transformation of the β-Li phase to a needle-like α-Mg phase. During the deformation process, the short-term diffusion of Mg atoms in the β-Li phase causes segregation at the large distortion, which leads to the phase transformation. The special shape of the α-Mg phase is caused by the activation of different slip systems. Therefore, the needle-like α-Mg phase in this paper may be caused by the free diffusion of Mg atoms in the β-Li phase during the solid solution process and segregation during the subsequent water quenching process, which leads to the phase transformation. 

### 3.2. Corrosion Properties of Mg-8.5Li-6.5Zn-1.2Y Alloys

#### 3.2.1. Hydrogen Evolution and Mass Loss

**Figure 5:**
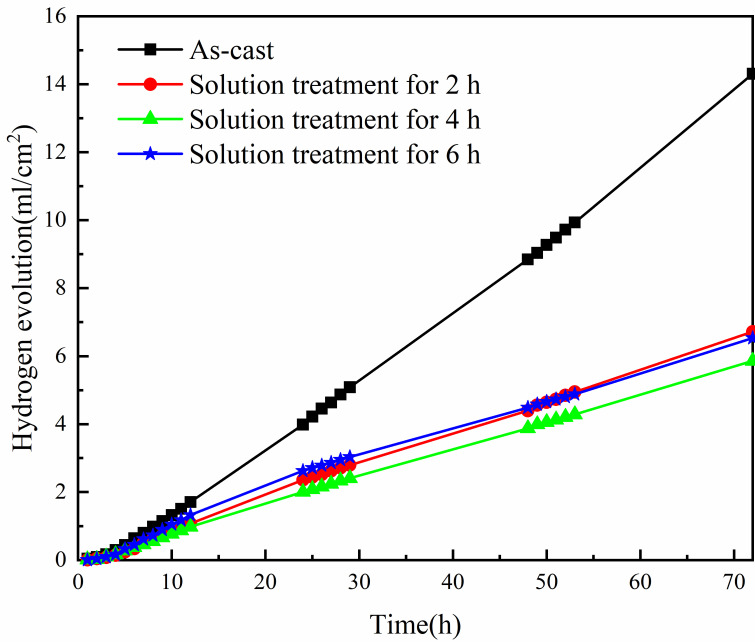
Hydrogen evolution curves of Mg-8.5Li-6.5Zn-1.2Y alloys with different solid solution treatment times measured in 3.5 wt.% NaCl solution.

The sample was soaked in 3.5 wt.% NaCl solution for 72 h at room temperature, and we recorded the volume of hydrogen collected by drainage method. We recorded the volume of hydrogen collected per hour for 12 h on the first day and recorded the volume of hydrogen collected per hour for 6 h on the second and third days, and evaluated the corrosion resistance of materials by calculating the rate of hydrogen evolution. Figure 5 shows the hydrogen evolution curves of the investigated Mg-8.5Li-6.5Zn-1.2Y alloys. It indicates that the hydrogen evolution rates of the Mg-8.5Li-6.5Zn-1.2Y alloy can be significantly reduced by solid solution treatment. The hydrogen evolution rate of the as-cast Mg-8.5Li-6.5Zn-1.2Y alloy is 14.31 mL·cm^−2^·h^−1^, which is the highest rate, and the hydrogen evolution rate of the Mg-8.5Li-6.5Zn-1.2Y alloy following solid solution processing for 4 h is the lowest rate, which is 5.86 mL·cm^−2^·h^−1^. The hydrogen evolution rates of the as-cast alloy after solid solution treatment for 2 h and 6 h are similar, which are 6.72 mL·cm^−2^·h^−1^ and 6.53 mL·cm^−2^·h^−1^, respectively. It is well known that the higher the hydrogen evolution rate, the worse the corrosion resistance of the material [9]. Therefore, it is evident that solid solution treatment can significantly improve the corrosion resistance of the Mg-8.5Li-6.5Zn-1.2Y alloy. As shown in Figure 5, by contrast, the alloy with solid solution treatment for 4 h has better corrosion resistance. 

**Figure 6:**
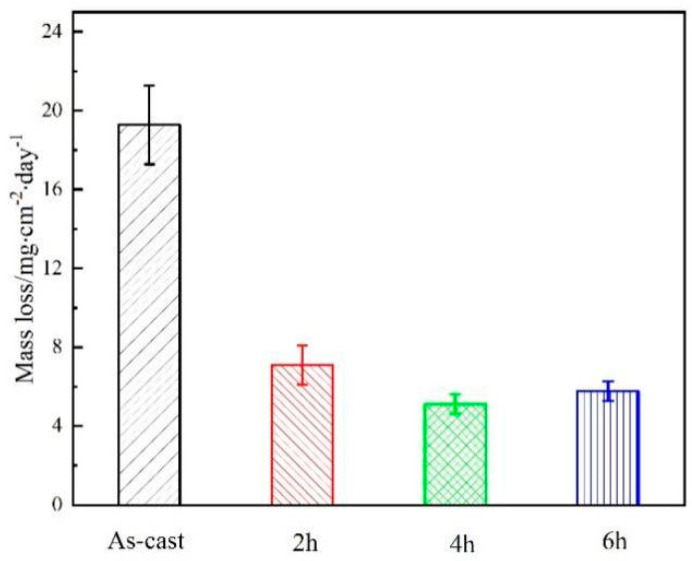
Mass loss data of the Mg−8.5Li−6.5Zn−1.2Y alloys with different solid solution treatment times measured in 3.5 wt.% NaCl solution.

Figure 6 shows the mass loss curves of the investigated Mg-8.5Li-6.5Zn-1.2Y alloys. After soaking the sample at room temperature for 48 h, the sample was taken out, rinsed, and dried with anhydrous ethanol. Then, the corrosion products on the sample surface were washed in a 180 g/L CrO_3_ chromic acid solution, and then rinsed and dried with anhydrous ethanol and weighed. The measured mass loss rate of the as-cast Mg-8.5Li-6.5Zn-1.2Y alloy is 19.28 mg·cm^−2^·d^−1^, which is calculated from Equation (1):

(1) $$v_{m}=\frac{m_{0}-m_{1}}{St}\;\;\;(\mathrm{mg}\cdot {\mathrm{cm}}^{-2}\cdot d^{-1})$$

The *v* is the average corrosion rate, *m*_0_ is the weight of the sample before corrosion, *m*_1_ is the weight of the sample after corrosion for a certain time and removal of corrosion products, *s* is the surface area of the specimen exposed to a corrosive solution, and *t* is corrosion time.

The mass loss rates of the samples with solid solution treatment for 2 h, 4 h, and 6 h are 7.09, 5.13, and 5.78 mg·cm^−2^·d^−1^, respectively. The results of the corrosion resistance of the material obtained from the mass loss experiment are in agreement with the results of the hydrogen evolution experiment: that is, solid solution treatment for 4 h > 6 h > 2 h > as-cast alloy. 

**Figure 7:**
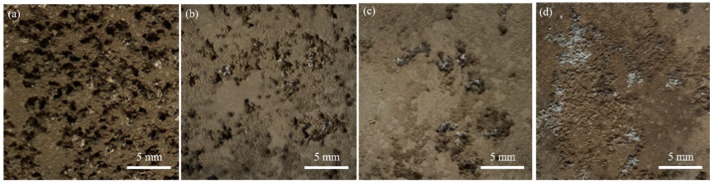
The surface morphology of the Mg-8.5Li-6.5Zn-1.2Y alloys with the different solid solution treatment times after mass loss experiment (**a**) as-cast, (**b**) 2 h, (**c**) 4 h, and (**d**) 6 h, respectively.

Figure 7 shows the surface morphologies of various samples after the mass loss experiment. The as-cast alloy is severely corroded, with many large and deep corrosion pits throughout the alloy surface (Figure 7a). In addition to the corrosion pits, there are many instances of transverse filiform-like corrosion penetrating the corrosion pits. Overall, the alloy surface is seriously damaged. After solid solution treatment, the amount of corrosion pits in the alloy decreases significantly. When the solid solution treatment time was less than 4 h, the corrosion pits decreased and the depth of the corrosion pits become shallower with the extension of solid solution treatment time, as shown in Figure 7b,c. When the solid solution treatment time is 6 h, the corrosion morphology is obviously different from other samples as shown in Figure 7d. It is evident that there is no deep corrosion pit on the surface and no obvious filiform corrosion characteristic. Although the growth of pitting pits and filiform corrosion are inhibited, the number of pitting pits obviously increases, distributed densely. This is perhaps connected to the special morphology of the α-Mg phase, as shown in Figure 3d.

#### 3.2.2. Electrochemical Measurement

**Figure 8:**
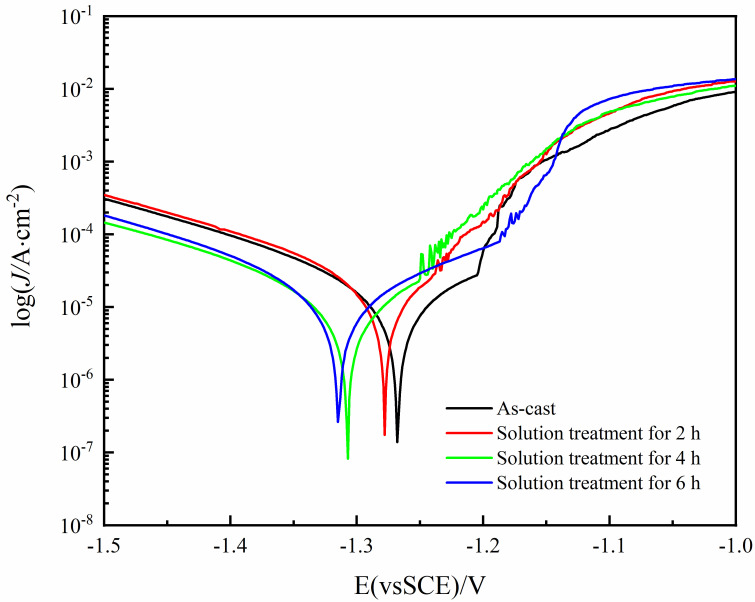
Potentiodynamic polarization curves of the Mg−8.5Li−6.5Zn−1.2Y alloys with the different solid solution treatment times measured in 3.5 wt.% NaCl solution.

The potentiodynamic polarization curves of the Mg-8.5Li-6.5Zn-1.2Y alloy investigated in 3.5 wt.% NaCl solution are shown in Figure 8. Because of the unique negative effect of the magnesium alloy [9], the polarization curve of the anode and cathode is asymmetric, and the increased rate of anode current density is much higher than that of the cathode current density. Cathodic branches in the potentiodynamic polarization curves of magnesium alloys correspond to the evolution of hydrogen, whereas anodic branches represent the dissolution of magnesium. [40]. Due to the negative difference effect and pitting corrosion, the anodic curve is usually not suitable for Tafel fitting analysis [41]. To ensure the accuracy and reproducibility of our tests, all tests were performed at least three times. The fitting data obtained by fitting the cathode curve of the polarization curve in this paper are shown in Table 1. As can be seen from Table 1, the corrosion potential (*E*_corr_) of the Mg-8.5Li-6.5Zn-1.2Y alloy in as-cast state and solid solution treatment for 2 h, 4 h, and 6 h are −1.268 V, −1.278 V, −1.308 V, and −1.315 V, respectively. The corrosion potential of the Mg-8.5Li-6.5Zn-1.2Y alloy is negatively shifted after solid solution treatment. In addition, the corrosion current density (*i*_corr_) values of the alloys are 3.40 × 10^−5^, 3.08 × 10^−5^, 1.98 × 10^−5^, and 2.26 × 10^−5^ A·cm^−2^, respectively. In general, high corrosion current density corresponds to fast hydrogen evolution reaction rate, which means poor corrosion resistance. Therefore, the alloy solid solution treatment for 4 h has the best corrosion resistance followed by solid solution treatment for 6 h and 2 h, and the corrosion resistance of the as-cast alloy is the worst.

**Figure 9:**
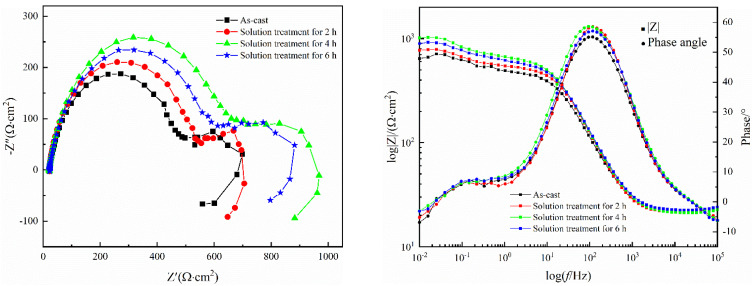
(**a**) Nyquist plots and (**b**) Bode plots of the Mg−8.5Li−6.5Zn−1.2Y alloys with the different solid solution treatment times measured in 3.5 wt.% NaCl solution.

EIS curves of the investigated Mg-8.5Li-6.5Zn-1.2Y alloys are shown in Figure 9. In the Nyquist plots, the curves are composed of a large loop with high-frequency capacitance, a small capacitive loop with medium frequency, and an inductive loop with a low frequency. The loop with high-frequency capacitance corresponds to the double electric layer capacitance formed between the sample surface and the solution. The semicircle in the medium frequency region corresponds to the barrier layer formed by the corrosion products. The presence of the inductive loop at the low frequency can be attributed to the pitting formed at the beginning of the local corrosion of the alloy [42]. It can be observed from the phase angle diagram that four samples have time constants in the high and low-frequency regions respectively, which also corresponds to the Nyquist plots. Generally, the more the capacitive loop’s radius increases, the better the corrosion resistance of the material. It is evident from the figure that the capacitive loop radius of the sample after solid solution treatment for 4 h is the largest, followed by 6 h and 2 h, and the as-cast alloy has the smallest capacitive loop radius, which is consistent with the results of the polarization curve. The impedance modulus |Z| value in the low-frequency region can directly reflect the sample’s resistance to corrosion during the test, and the larger the |Z| value, the better the corrosion resistance of the alloy. From Figure 9b, it can be found that the |Z| of the alloy in the low-frequency region (0.01 Hz) in the as-cast and different solution treatment times are 643.61, 771.61, 1016.3, and 894.86 Ω·cm^2^, respectively, which also indicates that the alloy has the best corrosion resistance for 4 h of solution treatment, which is in accordance with the above experimental results.

**Figure 10:**
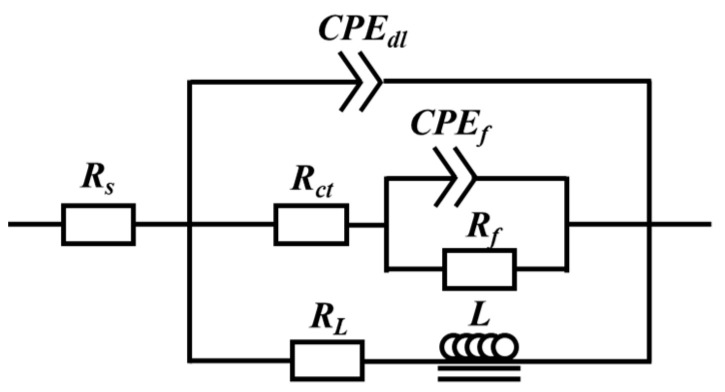
Equivalent circuits for the EIS of investigated alloys.

To examine the materials’ ability to resist corrosion further, the equivalent circuit diagram, as shown in Figure 10, was used to fit the EIS curves of the samples, and the fitted data are summarized in Table 2. In the equivalent circuit diagram, the solution resistance and charge transfer resistance, respectively, are denoted by R_s_ and R_ct_. The constant phase element CPE_dl_, which is defined by two values of Y_dl_ and n_dl_ (dispersion coefficient), is used to represent the electric double layer (interface between the electrode and electrolyte). CPE_dl_ denotes a resistance when n_dl_ is 0 and a capacitor when n_dl_ is 1. The film resistance and capacity are denoted by R_f_ and CPE_f_, respectively. For the low-frequency inductive loop, R_L_ and L stand for resistance and inductance, respectively. It suggests that localized corrosion has already started [43]. Usually, a high R_ct_ value indicates a high corrosion resistance of the material, the determined R_ct_ values of the Mg-8.5Li-6.5Zn-1.2Y alloy in as-cast state and solid solution treatment for 2 h, 4 h, and 6 h are 451.1, 499.6, 646, and 577.8 Ω·cm^2^, respectively. The fitting result is the same as the previous analysis result.

### 3.3. Corrosion Mechanisms

**Figure 11:**
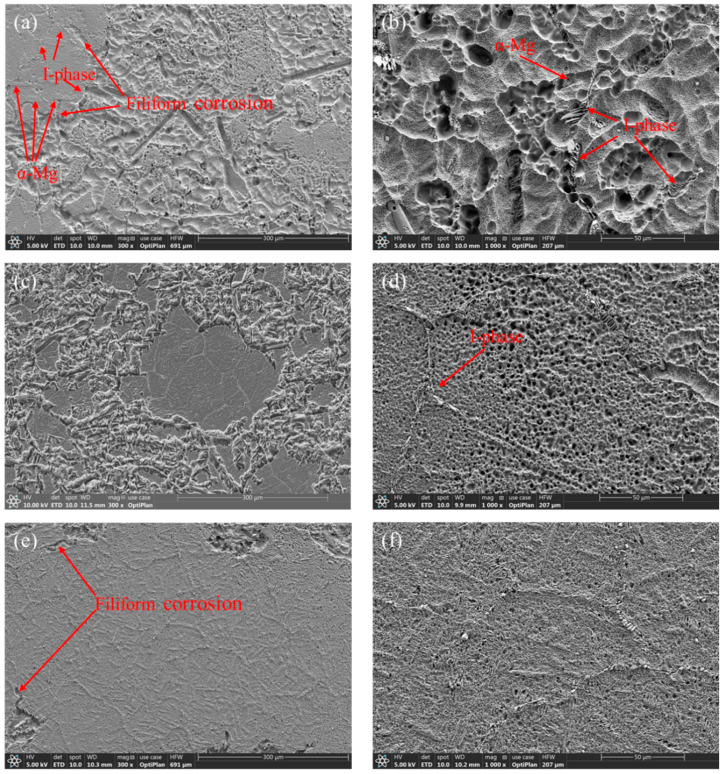

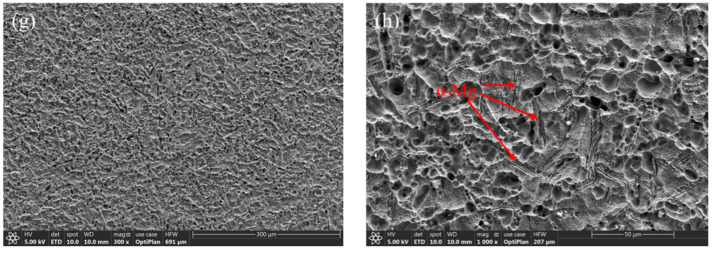
SEM images of the surface morphology of the Mg-8.5Li-6.5Zn-1.2Y alloys with the different solid solution treatment times after immersion for 2 h in 3.5 wt.% NaCl solution: (**a**) and (**b**) for as-cast; (**c**) and (**d**) for 2 h; (**e**) and (**f**) for 4 h; (**g**) and (**h**) for 6 h.

Figure 11 displays micrographs illustrating the morphology of the surface of the investigated Mg-8.5Li-6.5Zn-1.2Y alloys immersed in 3.5 wt.% NaCl solution for 2 h. The corrosion morphology of the four samples all show typical pitting corrosion and filiform-like corrosion characteristics, which is connected to the scenario where local cathodic and anodic sites change over time [43]. The surface of the as-cast alloy is corroded most seriously, and the corrosion surface of the alloy after 4 h solid solution treatment is the most complete. As shown in Figure 11a,e, when the filiform corrosion extends around the I-phase or α-Mg phase, it is suppressed by the I-phase or the boundary between the α-Mg and β-Li phases. In this respect, the I-phase and the boundary between α-Mg and β-Li are advantageous to the material’s ability to resist corrosion. However, a further magnifying view (Figure 11b,d,f,h) reveals that corrosion pits are mainly clustered around the α-Mg phase and I-phase. This is because the potential of the I-phase is higher than that of the α-Mg and β-Li phases [26], thus forming micro-galvanic corrosion with the matrix phase. In addition, the potential difference between the α-Mg phase and β-Li phase also causes galvanic corrosion [42]. 

As shown in Figure 11c,d, after solution treatment for 2 h, in contrast to the as-cast alloy, the corrosion pits are significantly smaller and the surface of the alloy is complete because the impurity phase and I-phase in the material are partially dissolved into the matrix to purify the matrix, as shown in Figure 3b. The impurity phase in the Mg-Li alloy is easy to form micro-galvanic corrosion with the matrix phase; moreover, the high potential impurity phase is protected as the cathode, while the low potential matrix phase is corroded as the anode. Therefore, although the reduction of the I-phase weakens the inhibition effect of corrosion, the risk of micro-galvanic corrosion in the matrix is greatly reduced, which enhances the material’s resistance to corrosion of the material compared with the as-cast alloy. After 4 h of solid solution treatment, the corrosion surface of the material is more complete. Moreover, the corrosion pits are the least and the filiform corrosion is effectively suppressed, as shown in Figure 11e,f. It can be explained that the I-phase precipitated again in the sample is distributed uniformly in the matrix (as shown in Figure 3c), giving full play to its role as a barrier to corrosion. In addition, the decrease of the α-Mg phase and impurity phases in the matrix reduces the corrosion probability of the material, so the samples solid solution treated for 4 h show the best corrosion resistance. After solid solution treatment for 6 h, the I-phase in the sample almost disappears, and the impact of the I-phase on the corrosion resistance can be negligible; however, the special microstructure of the α-Mg phase (Figure 3d) has a significant impact on corrosion. The needle-like α-Mg phase spreads throughout the matrix and forms micro-galvanic corrosion with the surrounding β-Li phase, making corrosion pits distributed all over the sample surface, as shown in Figure 11g. A further magnifying view (Figure 11h) reveals that, when the filiform corrosion is extended, it is blocked by the border dividing the α-Mg phase and β-Li phase. At the same time, due to the small size and uniform distribution of α-Mg, corrosion will occur from multiple locations in the matrix at the same time, and the oxide film is not easy to destroy, thus showing good corrosion resistance. Therefore, it is concluded that although the effect of the I-phase on corrosion is negligible, the special morphology of the α-Mg phase also improves the corrosion resistance of the alloy after solid solution treatment for 6 h. 

In general, although I-phase and the border dividing the α-Mg phase and β-Li phase will be corrosion breeding sites, they are more effective in inhibiting corrosion.

## 4. Conclusions

1. Generally, with the process of solid solution treatment, the amount of I-phase gradually decreases with the extension of the solid solution treatment time. Exceptionally, when the solution time is 4 h, the number of I-phases increases, and they are evenly distributed in the matrix. The amount of α-Mg phase decreases with the extension of solution time. When the solid solution treatment time reaches 6 h, the α-Mg phase is densely distributed in the matrix in the shape of a needle.
2. After solid solution treatment for 4 h, the precipitated I-phase can effectively hinder the expansion of filiform corrosion and the growth of pitting corrosion. Therefore, the material shows excellent corrosion resistance.
3. After solid solution treatment for 6 h, an abundance of phase boundaries are formed between the special acicular α-Mg phase and the β-Li phase, which become obstacles to corrosion and improve the corrosion resistance of the material.
4. The I-phase and α-Mg phase are the primary elements impacting the corrosion resistance of the Mg-8.5Li-6.5Zn-1.2Y alloy. On the one hand, the existence of the I-phase and the border dividing the α-Mg phase and β-Li phase can easily form galvanic corrosion, which has a bad effect on the corrosion resistance of the alloy. On the other hand, the I-phase and the border dividing the α-Mg phase and β-Li phase can hinder the corrosion of the alloy and improve the corrosion resistance of the alloy.

## Figures and Tables

**Table 1:** Fitting results from the polarization curves of the Mg-8.5Li-6.5Zn-1.2Y alloys with the different solid solution treatment times measured in 3.5 wt.% NaCl solution.

Alloys	*E*_corr_ (V_SCE_)	*i*_corr_ (A/cm^2^)	*β*_c_ (mV·dec^−1^)
As-cast	−1.268	3.40 × 10^−5^	−214.1
2 h	−1.278	3.08 × 10^−5^	−218.4
4 h	−1.308	1.98 × 10^−5^	−224.5
6 h	−1.315	2.26 × 10^−5^	−201.2

**Table 2:** Fitting results from the Nyquist plots of the Mg-8.5Li-6.5Zn-1.2Y alloys with the different solid solution treatment times measured in 3.5 wt.% NaCl solution.

Alloys	R_s_ (Ω·cm^2^)	Y_dl_ (Ω·cm^−2^ S^n^)	n_dl_	R_ct_ (Ω·cm^2^)	R_f_ (Ω·cm^2^)	L (H·cm^−2^)	R_L_ (Ω·cm^2^)
As-cast	21.51	3.31 × 10^−5^	0.87	451.1	1.77 × 10^2^	7.05 × 10^4^	563.21
2 h	21.37	2.52 × 10^−5^	0.90	499.6	1.84 × 10^2^	7.21 × 10^4^	702.37
4 h	21.20	3.02 × 10^−5^	0.87	646	2.21 × 10^2^	1.01 × 10^5^	884.52
6 h	22.45	2.80 × 10^−5^	0.87	577.8	2.02 × 10^2^	9.83 × 10^4^	814.35
